# What the South Carolina State Board of Health Proposes to Accomplish

**Published:** 1882-04

**Authors:** F. Peyre Porcher


### WHAT THE SOUTH CAROLINA STATE BOARD OF HEALTH PROPOSES TO ACCOMPLISH.

---

We will declare and define for all of our citizens what are legal nuisances, to be restrained or punished by law.

We will protect the people from the vicious practices of charlatans and empirics ; from those who would sell them poisons, adulterated and spurious drugs or articles of food.

We will go farther, and, like that fabled deity who eat his own offspring, we will see that their medical attendants are properly educated, morally and intellectually ; and shall not be permitted to offer their services as physicians unless possessed of a diploma from a respectable, legalized college.

We will increase the requirements for the education and licensing of druggist clerks, and give greater security in the preparation, sale, and dispensing of medicines, and thus lessen the number of those shocking accidents which are of such frequent occurrence.

We will see that those working signals on land and sea—switchmen and watchmen, steersmen and pilots—can tell yellow or blue from green, lest cars telescope or vessels collide, and a holocaust of victims be the penalty of defective vision in those, one of whose qualifications is to distinguish colors.

In conjunction with the National Board of Health, we will establish and define the intricate relations of, and hold plenary control over inter-state quarantine.

We will stamp out or eradicate, at their inception, the contagious diseases before they get a foothold in any community.

We will teach to our people that so fatal a malady as consumption is dependent upon soil-moisture—that great deduction of Bowditch!

We will invoke the aid of the civil engineer in the remodelling or reconstruction of the sewerage works of our cities and towns, and grant certificates to owners of houses which have been properly constructed as regards plumbing, ventilation, etc., which will increase their value for residence, for rental, or as investments.

We will also control the management of abattoirs, tanneries, manufacturing and other establishments, where injury to the people may ensue from imperfections or abuses in their operation.

We will seek to induce your honorable body to declare and define more precisely the rights of the citizen to create reservoirs, or to dam up or otherwise restrain the escape of water, thereby endangering the health of themselves or others.

If need be, we will create an abode for the reception and treatment of inebriates, and an additional asylum for the "incurable and harmlessly insane"—so as to relieve, in a measure, the demands made upon the State institution.

We will consider the propriety and feasibility of an attempt to limit the ravages of those diseases which, more than ever before, are sapping the life-blood of the nations—even to the third and fourth generations.
—*Dr. F. Peyre Porcher, "The Sanitary Needs of the State."*

